# Genetic Pathways and Functional Subnetworks for the Complex Nature of Bipolar Disorder in Genome-Wide Association Study

**DOI:** 10.3389/fnmol.2021.772584

**Published:** 2021-11-22

**Authors:** Chan-Yen Kuo, Tsu-Yi Chen, Pei-Hsiu Kao, Winifred Huang, Chun-Ruei Cho, Ya-Syuan Lai, Giou-Teng Yiang, Chung-Feng Kao

**Affiliations:** ^1^Department of Research, Taipei Tzu Chi Hospital, Buddhist Tzu Chi Medical Foundation, New Taipei, Taiwan; ^2^Department of Nursing, Cardinal Tien College of Healthcare and Management, New Taipei, Taiwan; ^3^Department of Emergency Medicine, Taipei Tzu Chi Hospital, Buddhist Tzu Chi Medical Foundation, New Taipei, Taiwan; ^4^Department of Emergency Medicine, School of Medicine, Tzu Chi University, Hualien, Taiwan; ^5^Department of Agronomy, College of Agriculture and Natural Resources, National Chung Hsing University, Taichung, Taiwan; ^6^School of Management, University of Bath, Bath, United Kingdom; ^7^Advanced Plant Biotechnology Center, National Chung Hsing University, Taichung, Taiwan

**Keywords:** genome-wide association study, pathway analysis, functional subnetwork, prior knowledge, bipolar, dementia

## Abstract

Bipolar disorder is a complex psychiatric trait that is also recognized as a high substantial heritability from a worldwide distribution. The success in identifying susceptibility loci for bipolar disorder (BPD) has been limited due to its complex genetic architecture. Growing evidence from association studies including genome-wide association (GWA) studies points to the need of improved analytic strategies to pinpoint the missing heritability for BPD. More importantly, many studies indicate that BPD has a strong association with dementia. We conducted advanced pathway analytics strategies to investigate synergistic effects of multilocus within biologically functional pathways, and further demonstrated functional effects among proteins in subnetworks to examine mechanisms underlying the complex nature of bipolarity using a GWA dataset for BPD. We allowed bipolar susceptible loci to play a role that takes larger weights in pathway-based analytic approaches. Having significantly informative genes identified from enriched pathways, we further built function-specific subnetworks of protein interactions using MetaCore. The gene-wise scores (i.e., minimum *p*-value) were corrected for the gene-length, and the results were corrected for multiple tests using Benjamini and Hochberg’s method. We found 87 enriched pathways that are significant for BPD; of which 36 pathways were reported. Most of them are involved with several metabolic processes, neural systems, immune system, molecular transport, cellular communication, and signal transduction. Three significant and function-related subnetworks with multiple hotspots were reported to link with several Gene Ontology processes for BPD. Our comprehensive pathway-network frameworks demonstrated that the use of prior knowledge is promising to facilitate our understanding between complex psychiatric disorders (e.g., BPD) and dementia for the access to the connection and clinical implications, along with the development and progression of dementia.

## Introduction

Many studies have suggested that there is a strong link between bipolar disorder (BPD) and dementia. BPD could increase the risk of developing some specific syndromes of dementia, especially for older adults (Masouy et al., 2011; Wu et al., 2013; Chen et al., 2015; Almeida et al., 2016; Diniz et al., 2017). Furthermore, Kessing and Andersen (2004) suggested that the rate of dementia is 6% higher for the patients with BPD who get admission to hospital with every episode than for those without BPD. BPD comes from a number of causes, such as ages, the duration of illness, polypharmacy, the presence of clinical comorbidity and so on (Borges et al., 2019). According to the Anatomical evidences, the gray matter volume and prefrontal cortex are both affected as people are suffered from BPD, and both of these two regions in the brain also have an influence on causing dementia (Pavlovic et al., 2011). On the one hand, the reduction of gray matter volume in the left cerebellar hemisphere and vermis volume increases the risk of dementia (Baldaçara et al., 2012). On the other hand, volumes of both hemispheres and the vermis are reduced when people suffered from BPD. Relatedly, Pavlovic et al. (2011) indicated that the dementia associated with BPD has a lot to do with psychosocial and functional impairment. Thus, dementia seems to be serious and inevitable. The significant symptom overlapping between dementia and psychiatric disorders like BPD is particularly an important therapeutic target with diagnostic challenges. Although clinical perspectives and implications with BPD and dementia were discussed previously (Lopes and Fernandes, 2012), the potential biologically functional pathways and molecular mechanisms still remains unclear.

Psychiatric traits are generally complex and multifactorial. Over the last decade, numerous genome-wide association (GWA) studies were conducted to search for susceptibility genes for complex human traits (Hindorffa et al., 2009). More than half or a few million markers in hundreds or thousands of subjects were conducted to increase the explanatory power of the disease heritability. A large number of low-risk genetic variants (usually odds ratios < 1.5) were identified to be involved in the etiology of complex traits (Manolio et al., 2008). However, the associated single-nucleotide polymorphisms (SNPs) and genes in total only account for a small proportion of the heritability for most of complex traits including BPD (Manolio et al., 2009). For instance, the effects of genes identified by linkage scans and association tests can only account for ∼2% of the ∼80% heritability of BPD (Crow, 2011). Many replication studies further demonstrated no replicable support for bipolar candidates (Crow, 2007). The failure in detecting true associations for heritable diseases like BPD might be involved with the “common-disease common-variant” hypothesis and the noise that is inherent in GWA studies and others (Maher, 2008; Gershon et al., 2011). We conducted allelic association tests for each SNP of three GWA datasets of BPD including the Wellcome Trust Consortium (WTCCC), the Genetic Association Information Network (GAIN), and the National Institute of Mental Health (NIMH). Again, only a few markers (*ATMIN*, *CENPN*, *HTR3B*, and *UBR1*) reached the commonly used genome-wide significance threshold level (*p* < 5 × 10^–8^) in the WTCCC GWA data, indicating the fact of potential noise inherent in genome-wide approaches. The noise may come from several sources such as small effect sizes at individual SNP level, causal variants (in particular when their minor allele frequency lower than genotyped SNPs) that are not in a complete linkage disequilibrium (LD) with SNPs, no power in inappropriate statistical methods, and others (Yang et al., 2010; Lee et al., 2011). In addition, due to the complexity of BPD, it is a challenge to identify which particular gene markers are the true causes of disease as noises may potentially be introduced due to technical or biological errors in nature (Ideker et al., 2011). This study aims to overcome these problems, in a gene-gene interaction sense, through identifying and finding the missing heritability.

In a GWA study, *p*-values are usually used to represent the statistical significance in the association, and the most significant SNP (*min-p*) of a gene region is selected to represent the significance level of a gene. However, the “*min-p*” approach is biased toward genes saturated with SNPs. Typically, large genes may have a higher gene-wise statistic, and in fact, we have previously observed a negative relationship between *p*-values and gene length (Yang et al., 2011; Kao et al., 2014). Introducing such bias into a subsequent pathway analysis may result in favoring pathways with larger genes. There are several Sidak’s correction based methods proposed to correct for a gene-size bias (Sidak, 1967; Saccone et al., 2007; Peng et al., 2010). In particular, a simple method based on the first order statistic (FOSCO) can well correct the gene-size bias by obtaining a gene-level significance for individual genes (Mirina et al., 2012). Although the FOSCO method does not deal with LD structures, its performance is as well as other methods such as GATES and VEGAS, whose computation is based on the LD structure (Mirina et al., 2012).

Bipolar disorder is a complex mental disorder with lifetime prevalence ranging from 8 to 5% in the general population and with a high probability of heritability around 80% (McGuffin et al., 2003; Kessler et al., 2005; Kato, 2007; Merikangas et al., 2008). Previous studies have also suggested the involvement of polygenic and multifactorial features in the pathology of BPD, along with the complex interactions among genes (G × G) and environmental (G × E) factors (Holmans et al., 2009; Pregelj, 2011; Chuang et al., 2013). Recently, we identified and prioritized candidate genes for BPD from multi-dimensional evidence-based data sources, which provide us an opportunity to explore an advanced pathway and network for BPD (Kao et al., 2014). With the combined scores obtained from the prior knowledge of BPD, each of the GWA genes was weighted by the magnitude of association to reduce noise (e.g., false-positive results and publication bias) and increase the effect size in pathway analysis (Pedroso et al., 2012). This hypothesis allows BPD candidates to play a larger role in pathways. The stronger the prior knowledge for BPD of a gene, the larger role the gene plays in pathways. Thus, these genes were regarded as “key genes.”

Genes normally cooperate with others having similar or related functions or characteristics to form a complex network of functional interactions to affect diseases, particularly for complex psychiatric traits. Genome-wide association studies provide the potential to account for such complexity. Thus, the pathway analytic strategy provides a basis of a gene-gene interaction to account for the biological relevance of genes and has the potential to detect the synergetic effects of multiple genes that might have been missed in the traditional single-marker association (Holmans, 2010; Fridley and Biernacka, 2011). The network analysis further provides a dynamic interrelationship among proteins to interconnect biological functions and molecular mechanisms, for instance, our previous work in major depressive disorder (Jia et al., 2011a). Most importantly, we want to know how genes aggregated into clusters of similar or related functions and how these components interconnect and function biologically in pathways and networks underlying the BPD. Therefore, pathway-based and network-based analyses are powerful approaches that summarize genetic information from sets of genes. Using such framework has the potential to interpret genes and pathways biologically. Thus, the objective of the present study uses the systems biology strategy to identify the missing heritability of BPD, which provides additional insights into the nature of complex genetic architecture underlying BPD.

**FIGURE 1:**
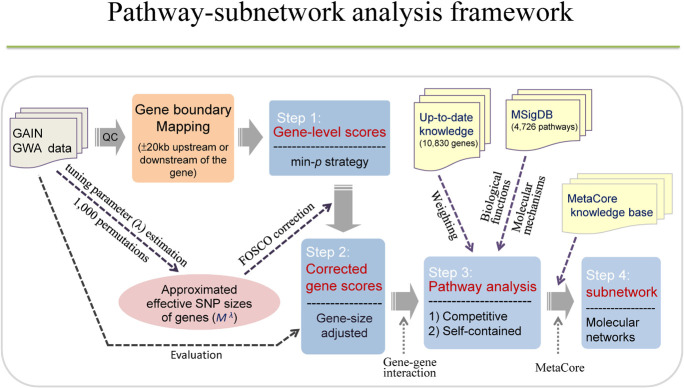
The study framework. The study consists of four steps, including calculation of gene-level scores, gene scores corrections, pathway analysis, and subnetwork analysis. Each gene was assigned a gene-level score using minimal *p*-value of association test among SNPs in a gene. Corrected gene scores can be obtained by calculating gene-size adjusted *p*-values based on FOSCO correction. Pathway analysis was conducted using competitive method (hypergeometric test, GSEA) and self-contained method (sum-statistic) with and without weighting scheme. Subnetwork analysis was performed to construct molecular networks using MetaCore.

Our current study intends to investigate enriched pathways and functional networks for BPD using a large-scale GWA dataset. We first conducted FOSCO method to correct for the gene-size bias by calculating the gene-level statistical significance. Second, we performed a pathway-based analysis using weighted competitive and self-contained methods with a minimum *p*-value approach to extract SNP information at a gene level. Third, we applied the subnetwork analysis to construct molecular networks. More importantly, the strategies used to conduct pathway or network-based analyses in the current study for bipolar potentially boosted our explanatory power to obtain meaningful results for studying the biological functions and molecular mechanisms of bipolar. For a more detailed study framework, please refer to Figure 1.

## Materials and Methods

### Genome-Wide Association Dataset

The BPD GWA dataset was accessed through the GAIN database of Genotypes and Phenotypes for bipolar disorders.^1^ A total of 1,001 bipolar cases and 1,034 healthy controls of Americans with European ancestry were included in this dataset. The genotyping platform was Affymetrix Genome-Wide Human SNP Array 6.0. After conducting quality control procedures (Manolio et al., 2007), a total of 698,227 SNPs were retained. We assigned a SNP to a gene if it was located within the gene or 20kb upstream or downstream of the gene. Therefore, a total of 416,371 SNPs were mapped into 15,213 protein-coding genes after dealing with aliases in the GAIN GWA dataset of BPD to perform pathway and network analyses. A basic allelic association test was used to calculate the genomic inflation factor for this GWA dataset, which was 1.03. The quantile-quantile plot for all analyzed SNPs can be found in Supplementary Figure 2, indicating a good quality of this GWA dataset.

### Bipolar Candidate Genes

We prioritized a list of 10,830 susceptible genes (Supplementary Data 1) that were collected from several lines of evidence-based datasets for BPD, including GWA study, association studies, linkage scans, gene expression (including human and animal studies), literature search, and biological regulatory pathways. For each gene, a dataset-specific score (*CS*_*j*_) was assigned in each data source according to the magnitude of association. All data types were combined using an optimized weighting vector to indicate the priority of the association of a gene with BPD. More detailed information of this gene prioritization procedure can be found in Kao et al. (2014).

### Pathway Annotations

To map genes into biological pathways, we used the Molecule Signature Database (MSigDB)^2^ annotations. The MSigDB consists of several open public sources of pathway annotations, including Gene Ontology (GO) terms, Kyoto Encyclopedia of Genes and Genomes (KEGG), BioCarta, Reactome, and gene sets compiled from published biomedical literature (Subramanian et al., 2005), which listed 4,726 pathways and 22,429 genes. Pathways with extreme numbers of genes (i.e., 10th percentile of pathway-size distribution, <10 or >380 genes) were removed from analyses to avoid stochastic bias or testing any over-general biological process. This procedure resulted in a total of 4,120 pathways left in the GAIN GWA dataset.

### Gene-Wise Statistical Significance Correction of Gene-Size Bias

To obtain a gene-level statistical significance, we first mapped SNPs to a gene (using NCBI build 36) if SNPs were located within the gene region or 20 kb upstream or downstream of the gene, which was suggested as a good gene boundary (Jia et al., 2011b). We used a commonly adopted method to select the most significant SNP (*min-p*, denoted as *p*^*min*^) among *M* SNPs in a gene region in association tests to represent the significance level of a gene. Because the *p*-values are biased toward to a gene-length, we utilized *p*^*adj*^ = 1 − (1 − *p*^min^)^*M*_*eff*_^ to adjust it to the gene-wise statistical significance. We approximated the effective number, or alternatively adjusted number, of SNPs (*M*_*eff*_) using *_*M*_*^λ^ to correct for the actual number of SNPs (*M*), where the tuning parameter λ satisfies the correlation between adjusted *p*-values (*p*^*adj*^) and *M* is minimal; that is, min|corr(*p*^*adj*^, *M*)|. The value of the tuning parameter λ can be optimized empirically on permuted genotype data under the null through randomly permuting case/control status of subjects, keeping the genotypes remain the same. If *p*^*adj*^ are well corrected for the gene-size, they would be uniformly distributed from [0,1]. For a detailed method, please refer to Mirina et al. (2012).

### Statistical Methods for Pathway Enrichment Analysis

We applied three statistical methods to test the enrichment of significant pathways for BPD. According to prior studies, we extended two permutation-based approaches, the Gene Set Enrichment Analysis (GSEA, a competitive method) and the sum-statistic method (a self-contained method), by taking into account prior knowledge on BPD (Wang et al., 2010, 2011). We denoted *D* as the disease of interest (here is BPD), and *r*_*j*_(*D*) as the gene-wise statistic value that defined as the logarithm of adjusted gene-wise *p*-values of the corresponding to the most significant SNP in gene *j*(*j* = 1, …, *N*). Here we allowed bipolar candidate genes to play a larger role in pathway analyses. A weight (≥1), $w_{j}=1+\frac{CS_{j}}{\bar{CS}}$, proportional to the prior knowledge (i.e., magnitude of association) is particularly assigned to gene *j*, where $\bar{CS}$ represents the mean of combined scores of all bipolar candidate genes. Thus, a weighted GSEA (*w*GSEA) was generalized. A set of genes (*g*) was first ordered according to the weighted gene-wise statistic values [*w_*j*_r_*j*_*(*D*)] so that genes with a stronger significance (or small *p*-values) are ranked on the top. For each tested pathway (*S*), an enrichment score (ES) was calculated based on *p*-values of a gene-set in each pathway. The ES can be written as $ES=\max_{1\leq j\leq N}\left\{\sum_{g\in S,j\leq i}\frac{{\left|w_{j}r_{j}\left(D\right)\right|}^{p}}{N_{R}}-\sum_{g\notin S,j\leq i}\frac{1}{N-N_{H}}\right\}$ which consists of two parts, namely, gain (if gene is in a pathway) and loss (if gene is not in a pathway), where *N*_*H*_ represents the number of genes in a pathway *S* and *N*_*R*_ = ∑_*g* ∈ *S*_|*w*_*j*_*r*_*j*_(*D*)|^*p*^ is the total gain with *p* = 1. The ES was used to evaluate association signals for each annotated pathway. Then, for each pathway, the ES was normalized to compute NES by subtracting the mean of the ES in the permutated data sets, *ES*(*S*^*perm*^), and divided by the standard deviation of *ES*(*S*^*perm*^). We calculated empirical *p*-values for all pathways using 5,000 permutations to compare the original ES score from the GWA dataset and the permutation datasets (denoted as *S*^*perm*^) by computing the fraction of the numbers of {*ES*(*S*^*perm*^) > *ES*(*S*)} divided by the total number of permutations. In a weighted sum-statistic (*w*SS) method, only genes in a specific pathway were considered, while part of those genes may play a larger role in the pathway. The *w*SS method calculates the sum of the weighted gene-wise statistic values over the set of genes $\left(\sum_{j=1}^{k}w_{j}r{\left(D\right)}_{j}\right)$. Alternatively, a statistical probability hypergeometric model was applied. In the hypergeometric test, we used a cutoff *p*-value of 0.05 to define significant genes using their gene-wise statistics (i.e., *p-*values). A *p*-value based on a hypergeometric distribution for each pathway was computed to describe the probability of interest genes (i.e., significant genes) in a specific pathway without a replacement from the whole GWA genes. We performed the hypergeometric test for all annotated pathways using the GWA dataset for BPD.

### Biologically Functional Subnetwork Analysis

To perform the biologically functional subnetwork analysis, we selected genes from 15,213 GAIN GWA genes only if the gene contains at least one SNP having gene-wise statistic *p*^*adj*^ < 0.05 and the gene provides prior knowledge (i.e., having combined score greater than the total mean of combined scores) as these genes were denoted as seed genes for a further subnetwork analysis. We applied the AUTO expand algorithm in software, MetaCore^3^, to these seed genes. A large network was constructed to the initial list of seed nodes (i.e., seed genes). Then, we cut the large network into several subnetworks according to the following procedures. Firstly, we expanded edges from the most relevant nodes (i.e., proximity of a node and traffic/flow through the node) for the outgoing (•→) path direction. Secondly, a flow value was calculated for each of seed nodes, with the flow through it equal to 1, according to algorithm. For example, a node has three incoming flows (each with flow value of ¼), and then the node receives a flow value of ¾. On the contrast, if the sum of incoming flows exceeds 1, the resulting flow value will be reduced to 1. Thirdly, we only considered the most connected node and selected the nodes that have the highest flow values. Fourthly, we iterated the process until the included nodes exceeded a default limit of 50. Fifthly, we applied the above steps for ingoing (←•) path direction and merged them into one subnetwork. Sixthly, the nodes selected for the subnetwork from the large network were deleted. Finally, a new subnetwork was reconstructed until no more subnetworks can be generated.

Each subnetwork provides a Z-score that ranks the subnetworks according to their saturation with genes from the initial list of seed nodes. The formula for the Z-score is


$$Z-score=\frac{r^{node}-n^{node}\left(\frac{R^{object}}{N^{node}}\right)}{\sqrt{n^{node}\left(\frac{R^{object}}{N^{node}}\right)\left(1-\frac{R^{object}}{N^{node}}\right)\left(1-\frac{R^{object}-1}{N^{node}-1}\right)}},$$

where *r*^*node*^ and n*^*node*^* represent the number of nodes and the total number of nodes in each subnetwork generated from the seed nodes, respectively, *R*^*object*^ represents the number of network objects corresponding to the genes and proteins in the seed nodes, and *N*^*node*^ represents the total number of nodes in MetaCore^TM^ database. A high Z-score exhibits that the network is highly saturated with genes from the seed genes. Similar to Z-score, we compared the genes in the subnetwork versus the genes not in the subnetwork within the full set of all genes (i.e., MetaCore base knowledge) on maps, and calculated a *p*-value based on the hypergeometric distribution for each subnetwork to estimate the probability for a particular mapping to a subnetwork.

### Multiple Testing Corrections

To account for multiple testing problems in the pathway and network analyses, we used the method proposed by Benjamini and Hochberg (1995) to control for the false discovery rate (FDR). We ordered all the *p*-values of pathways and compared each *p*-value *p*(*i*) with a threshold of (*i*/*m*)*q*^∗^, where *m* represents the total number of pathways, and *q*^∗^ represents the significance level. Thus, the procedure controls for the FDR at *q*^∗^ = 0.05 level in this current study, assuming *p*-values are independently distributed under null hypotheses.

## Results

**FIGURE 2:**
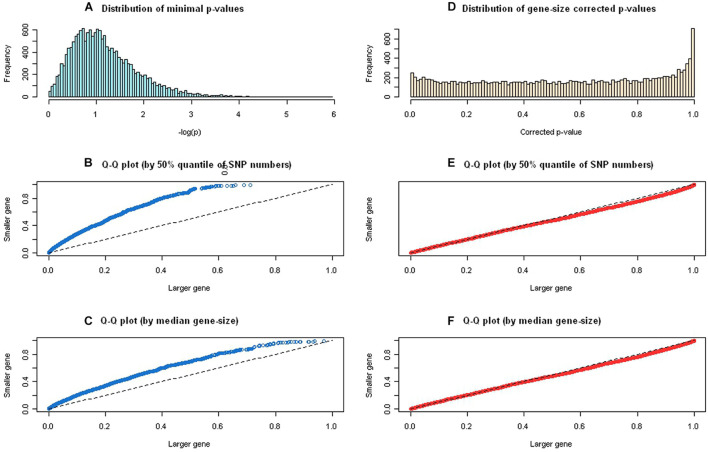
The distribution of minimal *p*-values and quantile-quantile plots before and after gene-size correction. **(A)** Distribution of logarithm of minimal *p*-values was skewed to the right. **(B)** The quantile-quantile plot, using 50% quantile of single-nucleotide polymorphism (SNP) numbers, showed a significant correlation between minimal *p*-values and the gene-size. **(C)** The quantile-quantile plot, using median gene size, showed a significant correlation between minimal *p*-values and the gene size. **(D)** Distribution of logarithm of gene-size corrected *p*-values demonstrated uniformly distributed from [0,1]. **(E)** The quantile-quantile plot, using 50% quantile of SNP numbers, showed no significant correlation between gene-size corrected *p*-values and the gene-size. **(F)** The quantile-quantile plot, using median gene size, showed no significant correlation between gene-size corrected *p*-values and the gene size.

A total of 416,371 SNPs were annotated and mapped into 15,213 protein-coding genes in the GAIN GWA study of BPD, which were then mapped to 4,726 annotated pathways in the gene-pathway mapping process. We computationally optimized the tuning parameter λ in the gene-wise statistical significance correction step, and the value 0.85 was estimated iteratively to approximate the effective number of SNPs (see Supplementary Figure 2) for calculating gene-size corrected *p*-values using the GAIN GWA study for BPD. Figure 2 displays the distribution of minimal *p*-values and quantile-quantile plots before and after the gene-size correction. We used 50% quantile of SNP numbers (i.e., >13 SNPs) and median of gene lengths (i.e., >23.15mb) to define a large gene. The distribution of logarithm of minimal *p*-values was skewed to the right (Figure 2A) and the quantile-quantile plots were far away from the 45° line (i.e., under null hypothesis of no correlation), which showed a significant correlation (*p* = 2.2 × 10^–16^) between minimal *p*-values and the gene-size (Figures 2B,C). After adjusted for the gene-length, the corrected *p*-values approximated uniformly distributed from [0,1] (Figure 2D) and their quantile-quantile plots followed the 45° line, which exhibit no any correlation (*p* = 0.13) between corrected *p*-values and the gene-size (Figures 2E,F).

In total, 87 enriched pathways (see Supplementary Table 1) were identified for their biological relevance in BPD using the GAIN GWA dataset after controlling the FDR at the 0.05 level. Table 1 summarizes 36 significant pathways that were simultaneously enriched in both with or without weighting schemes under competitive methods (*w*GSEA and hypergeometric test) and self-contained method (*w*SS). Of which, 26 pathways were identified in permutation-based approaches (i.e., 22 out of 80 were identified in GSEA and 12 were identified in Sum-statistic, with 8 overlaps). The eight overlapping pathways (six from KEGG and two from Reactome) are drug metabolisms of other enzymes, pentose and glucuronate interconversions, starch and sucrose metabolism, ascorbate and aldarate metabolism, retinol metabolism, porphyrin and chlorophyll metabolism, glucuronidation, and phase II conjugation, which related to drug metabolism, carbohydrate metabolism, metabolism in cofactors and vitamins, xenobiotic metabolism, immune system, cell differentiation, cellular communication, cellular signal transduction, and growth factors. The remaining 18 pathways (3 from KEGG, 6 from GO, 2 from Reactome, and 7 from Curated gene sets) are mainly involved with lipid metabolism, xenobiotics biodegradation and metabolism, ion transport, molecular transport, cellular component, cellular communication, cell differentiation, immune system, growth factors, and oncogenes and translocate cancer genes. From a statistical and probabilistic point of view, 10 enriched pathways (7 from GO and 3 from Curated gene sets) were found significant using the hypergeometric test. Those pathways were structurally mapped to channel activities (i.e., voltage-gated channel activity, gated channel activity, voltage-gated cation channel activity and cation channel activity), molecular transport activities (i.e., cation or ion transmembrane transporter activity and metal ion transmembrane transporter activity), immune system, cell differentiation, cellular communication, cellular signal transduction, transcription factor, and growth factor.

**TABLE 1:** Significantly enriched pathways in the Genetic Association Information Network (GAIN) genome-wide association (GWA) study for bipolar disorder (BPD) using competitive and self-contained methods with and without weighting scheme.

			Permutation-based								Probability-based	
			GSEA				Sum-statistic				Hypergeometric test	
Annotated pathway^a^	$n_{\text{GWA}}^{\text{pw}}/n^{pw}$	$n_{\text{BPD}}^{\text{pw}}$	Equal weight		Weighting		Equal weight		Weighting			
					
			ES	*p*_*BH*_ ^*b*^	ES	*p*_*BH*_ ^b^	SS	*p*_*BH*_ ^b^	SS	*p*_*BH*_ ^b^	*p*_*nom*_	*p*_*BH*_ ^b^
**KEGG:**												
**Drug metabolism other enzymes**	48/51	26	0.68	**<10^–4^**	0.72	**<10^–4^**	42.24	**<10^–4^**	73.01	**<10^–4^**		
**Retinol metabolism**	60/64	42	0.67	**<10^–4^**	0.66	**<10^–4^**	53.94	**<10^–4^**	93.48	**<10^–4^**		
**Pentose and glucuronate interconversions**	25/28	13	0.73	**<10^–4^**	0.78	**<10^–4^**	28.58	**<10^–4^**	50.73	**<10^–4^**		
**Porphyrin and chlorophyll metabolism**	37/41	19	0.69	**<10^–4^**	0.75	**<10^–4^**	31.95	**<10^–4^**	57.27	**<10^–4^**		
**Starch and sucrose metabolism**	49/52	32	0.63	**<10^–4^**	0.70	**<10^–4^**	38.81	**<10^–4^**	70.11	**<10^–4^**		
**Ascorbate and aldarate metabolism**	23/25	12	0.79	**<10^–4^**	0.81	**0.03**	27.54	**<10^–4^**	50.67	**<10^–4^**		
Drug metabolism cytochrome P450	68/72	54					53.22	**<10^–4^**	98.54	**<10^–4^**		
Metabolism of xenobiotics by cytochrome P450	68/70	54					51.41	**<10^–4^**	94.61	**<10^–4^**		
Steroid hormone biosynthesis	51/55	36					38.49	**<10^–4^**	69.22	**<10^–4^**		
**GO:**												
Extracellular region part	313/332	218	0.49	**<10^–4^**	0.55	**<10^–4^**						
Extracellular space	224/239	155	0.51	**<10^–4^**	0.57	**<10^–4^**						
Ion transport	174/184	108	0.54	**<10^–4^**	0.54	**0.05**						
Substrate specific transmembrane transporter activity	321/341	207	0.49	**<10^–4^**	0.51	**0.05**						
Substrate specific transporter activity	366/388	236	0.48	**<10^–4^**	0.50	**0.05**						
Cytosol	191/202	139	0.50	**0.03**	0.55	**0.03**						
Cation transmembrane transporter activity	201/211	130									1.2 × 10**^–^**^6^	**0.002**
Ion transmembrane transporter activity	259/275	169									6.7 × 10**^–^**^6^	**0.006**
Metal ion transmembrane transporter activity	140/145	91									7.3 × 10**^–^**^6^	**0.006**
Voltage-gated channel activity	70/73	46									3.1 × 10**^–^**^5^	**0.02**
Gated channel activity	115/121	76									3.8 × 10**^–^**^5^	**0.02**
Voltage-gated cation channel activity	64/66	41									5.8 × 10**^–^**^5^	**0.03**
Cation channel activity	115/118	78									6.3 × 10**^–^**^5^	**0.03**
**REACTOME:**												
**Glucuronidation**	17/19	8	0.83	**<10^–4^**	0.81	**<10^–4^**	22.50	**<10^–4^**	37.16	**<10^–4^**		
**Phase II conjugation**	56/60	35	0.60	**<10^–4^**	0.66	**0.03**	42.18	**<10^–4^**	72.53	**<10^–4^**		
Purine ribonucleoside monophosphate biosynthesis	11/11	6	0.82	**0.03**	0.84	**0.05**						
Biological oxidations	120/127	83					76.62	**<10^–4^**	133.5	**<10^–4^**		
**Curated gene-set:**												
Riggi ewing sarcoma progenitor UP	375/426	245	0.46	**<10^–4^**	0.54	**<10^–4^**						
Zhang breast cancer progenitors UP	360/448	215	0.46	**<10^–4^**	0.49	**0.03**						
Mullighan mll signature 1 UP	351/389	235	0.46	**0.02**	0.50	**0.03**						
Weber methylated icp in fibroblast	16/16	6	0.76	**0.02**	0.75	**0.03**						
Rizki tumor invasiveness 3D DN	215/234	145	0.49	**0.02**	0.53	**0.05**						
Bonci targets of MIR15A and MIR16_1	81/81	57	0.57	**0.04**	0.61	**0.05**						
Mccabe bound by HOXC6	350/461	228	0.46	**0.05**	0.50	**0.05**						
Martinez response to trabectedin	40/42	27									5.9 × 10**^–^**^10^	**2.4** × **10^–6^**
Manalo hypoxia UP	191/210	127									6.5 × 10**^–^**^6^	**0.006**
Onder CDH1 targets 2 UP	226/258	159									3.0 × 10**^–^**^5^	**0.02**

*n^pw^, the number of genes in pathway; $n_{GWA}^{pw}$, the number of genes on chip; $n_{BPD}^{pw}$, the number of bipolar candidate genes in pathway (i.e., prior knowledge to BPD); GSEA, gene-set enrichment analysis; ES, enrichment score; SS, sum-statistic; p_nom_, nominal p-value.*

*^a^ GSEA and Sum-statistic results were based on 5,000 permutations and hypergeometric test results were based on statistical probability model.*

*^b^ The p_BH_ were based on Benjamini and Hochberg (1995) multiple testing correction.*

*Pathways with p-values highlighted in bold are significantly enriched for their biological relevance in BPD.*

We selected 274 genes (denoted as seed nodes) that show a high chance to associate with BPD (see our selection criteria in Materials and methods) from 15,213 GAIN GWA genes further for a functional subnetwork analysis. The selection of the 274 seed nodes was unlikely to be affected by large genes (correlation coefficient = −0.045, *p* = 0.46). The type and location of the 274 seed nodes were summarized in Table 2. These genes were mainly allocated to G-protein coupled receptor (e.g., *GRM1* and *ADRA1B* in plasma membrane), growth factor (e.g., *FGF5* and *TGFA* in extracellular space), ion channel (e.g., *KCNB1* and *CACNA2D* in plasma membrane; *ITPR2* and *NOX5* in cytoplasm), ligand-dependent nuclear receptor (e.g., *NR3C2* in nucleus), transcription regulator (e.g., *PAX* in nucleus; *WHAH* in cytoplasm), transmembrane receptor (e.g., *IL17RA* in plasma membrane; *TSPO* in cytoplasm), transporter (e.g., *ATP6V1B2* in cytoplasm; *SLC16A4* in plasma membrane), and others.

**TABLE 2:** Type and location of the 274 seed nodes^*a*^ selected from the GAIN GWA genes.

Type	Location	Genes^*b*^
G-protein coupled receptor	Plasma membrane	*ADRA1B, BAI1, GPR133, GRM1, NMBR, OR2B6, OR9G1, PTGER4*
Growth factor	Extracellular space	*FGF5, NELL1, TGFA*
Ion channel	Plasma membrane	*ACCN3, CACNA2D, CNGA3, KCNB1, KCNIP1, KCNQ1, KCNS1, PKD2L1, SCN3B, TRPC7, TRPV1, TRPV3*
	Cytoplasm	*ITPR2, NOX5, PEX5L*
	Unknown	*NALCN*
Ligand-dependent nuclear receptor	Nucleus	*NR3C2*
transcription regulator	Nucleus	*ADNP, ANKRD55, ATF3, ATF4, BASP1, CDYL, CREBBP, DMRT1, DNMT3L, FANK1, FOXM1, HIF3A, HIVEP2, HNF1B, KIDINS220, MLL4, NCOA1, PAX5, REST, RFX2, SIM1, TCF7L1, VSX1, ZBTB32, ZSCAN2*
	Cytoplasm	*ATF6Y, WHAH*
Transmembrane receptor	Plasma membrane	*CD40, GFRA1, IFNGR1, IL17RA, KIR2DS4, LMBR1, SFRP1, TYROBP*
	Cytoplasm	*TSPO*
Transporter	Cytoplasm	*ABCB8, ATP6V1B2, CCT6B, DOC2A, FABP3, SLC25A13, SYT2, SYT6, VPS26B*
	Plasma membrane	*ATP10B, SLC16A4, SLC17A3, SLC2A13, SLC4A10, VTI1A SLC5A1, SLC5A7, SLC6A15, SORCS2, STXBP3, TM9SF2*,
	Unknown	*SLCO5A1, SYT14*
Peptidase	Cytoplasm	*GZMB, PEPD*
	Extracellular space	*HABP2, MMP7*
	Plasma membrane	*DPP4*
	Unknown	*USP13*
Phosphatase	Plasma membrane	*PPFIA2, PTPRB*
	Cytoplasm	*PPM1A*
Chemical- endogenous mammal	Unknown	*C3*
Cytokine	Extracellular space	*CCL1, IFNA7, IFNG, IL26, WNT2, WNT4*
Enzyme	Cytoplasm	*ADSL, ALDH9A1, BRAF, BTRC, CNOT4, CYP26A1, CYP2C18, FBXL2, GCLM, GM2A, GNPAT, HEXA, HS3ST5, HSP90AA1, IRS1, MGAT4A, MTHFD1L, NAT1, NOS3, PAFAH1B2, PDHX, PLCG2, RAB21, RAB2A, RAB6A, TIAM2, UGT1A1, UGT1A4, UGT1A10, UGT1A8*
	Plasma membrane	*ADCY6, DAGLA, FADS1, GNG7, RAB4B, VNN1*
	Nucleus	*CA9, FNBP1, MAD2L2, SMARCAD1, SMYD3, TARS*
	Extracellular space	*SOD3*
	Unknown	*FAM135A, LCMT2, MBOAT2*
Kinase	Cytoplasm	*CHUK, CSNK1E, DCLK2, DGKH, EIF2AK4, PRKCE, STK4, TAOK2*
	Nucleus	*CDK5, CDK6, VRK2*
	Plasma membrane	*MAGI2, TRAT1, TRIO*
	Unknown	*RBKS, TTBK2*
Other	Cytoplasm	*ADAL, ARC, ARRB1, BAG1, BCAS4, BLNK, CIDEA, CST5, EFHA1, EML1, FAM129B, HBXIP, HSPB6, MFHAS1, NLRP5, NOS1AP, RASGRP3, RIN2, RPS7, SOCS6, STARD10, STARD13, STMN4, SWAP70, TBC1D15, TPM2*
	Extracellular space	*COL28A1, FBLN2, FST, LAMA5, NLRP7, TIMP2*
	Nucleus	*BCL11B, BMS1, CABLES1, CDAN1, FYB, GCKR, HIRIP3, HIST1H2AB, HIST1H3B, HIST1H4B, JRK, MAD1L1, NCAPD3, PCBP3, RANBP3, RASSF2, RBM15, SNRPA, SFRS5, SYNE1, ZFP161, ZMYND11, ZNF260, ZNF295, ZNF461, ZNF564, ZNF592*
	Plasma membrane	*ANK3, BTN2A1, CD276, CD53, CDH19, CDH22, CDH4, CGNL1, DSC3, EPB41L5, KIAA1797, GYPA, NPTN, PLXDC1, RPH3AL, SEZ6L, SGCG, SIRPB1, STOM, TANC1, TLN1, TSPAN15*
	Unknown	*ACTR3B, ADAMTSL3, BICC1, CACNA2D4, CALM3, CCDC122, CDKAL1, CTDSPL2, DNHD1, DPY19L1, FHAD1, GRAMD1B, KRTAP4-5, LONRF1, ODZ4, PHF21B, PSAPL1, SLC38A10, TMEM133, TMEM51, TMEM54, TTC15, TRIM38, TTC39B, TTLL12, WDFY2*

*^a^ All selected genes were mapped in the Ingenuity Pathways Analysis (IPA).*

*^b^All genes contained at least one SNP having gene-wise statistic p^adj^<0.05 and provided information of combined scores greater than the median. Detailed methods of calculating combined scores please refer to Kao et al. (2014).*

**FIGURE 3:**
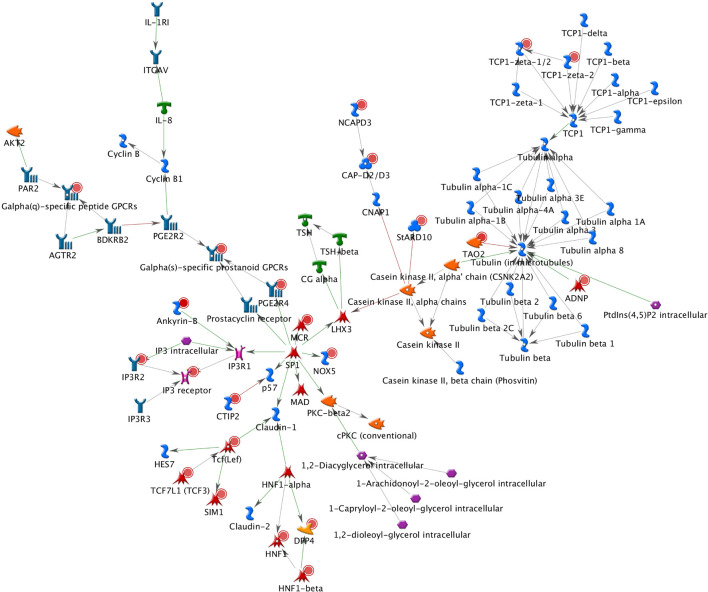
The top one functional subnetwork. This subnetwork was saturated with 22 objects and 39 interactions, with a hub in transcription factor SP1 with 10 activation interactions. Thick cyan lines indicate the fragments of canonical pathways. Upregulated genes are marked with red circles and downregulated with blue circles. Green and red arrows indicate activation and inhibition effect, respectively.

**FIGURE 4:**
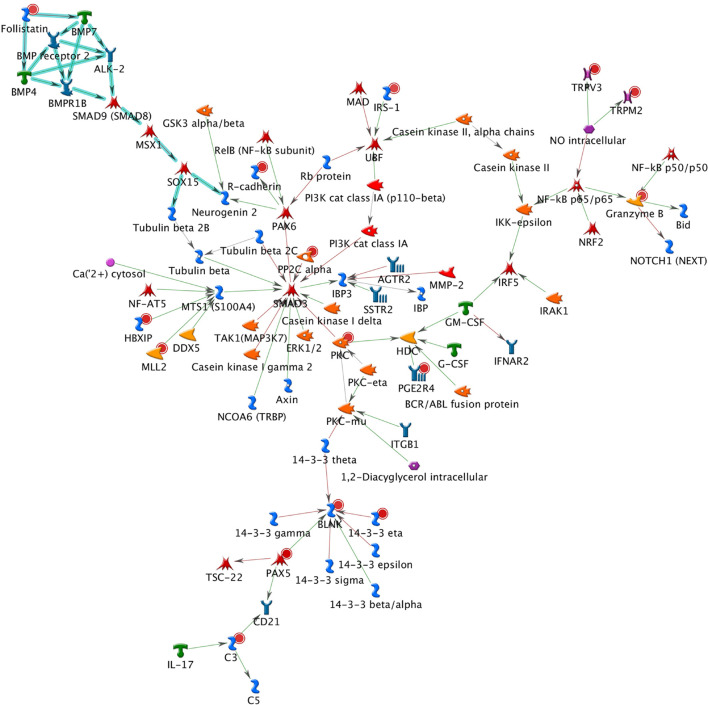
The top two functional subnetwork. This subnetwork contained 15 objects and 83 interactions, which centered around six hubs, including three transcription factors (SMAD3, PAX6, and UBF), two generic binding proteins (BLNK and MTS1), and one generic enzyme (HDC). Thick cyan lines indicate the fragments of canonical pathways. Upregulated genes are marked with red circles and downregulated with blue circles. Green and red arrows indicate activation and inhibition effect, respectively.

**FIGURE 5:**
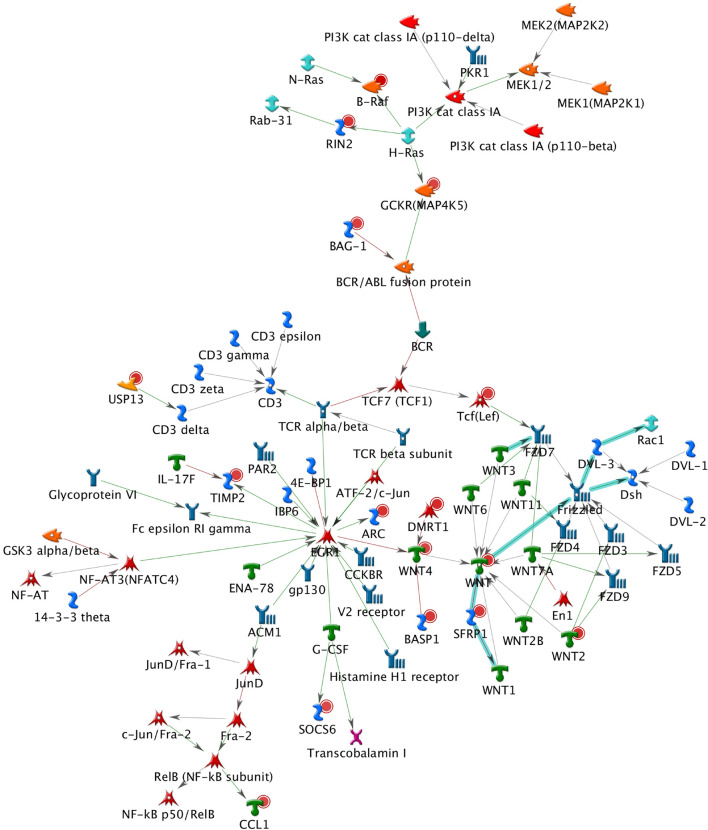
The top three functional subnetwork. This subnetwork contained 16 objects and 64 interactions, with two hubs in a transcription factor (EGR1) and a GPCR receptor (FZD7) with ranging from 5 to 18 interactions. Thick cyan lines indicate the fragments of canonical pathways. Upregulated genes are marked with red circles and downregulated with blue circles. Green and red arrows indicate activation and inhibition effect, respectively.

A total of 26 subnetworks were constructed in MetaCore using these 274 seed nodes. The crosstalk information and statistical tests for network saturation of the top three function-related biological subnetworks were listed in Table 3 and the remaining subnetworks in Supplementary Table 2. The top one functional subnetwork (Figure 3) was saturated with 22 objects (spanned by 15 seed nodes) and 39 interactions (33 were activation and 6 were inhibition), which has a hub in transcription factor SP1 with 10 activation interactions (*p* = 2.33 × 10^–20^, Z-score = 24.35). This subnetwork was involved with several GO processes such as *de novo* posttranslational protein folding, *de novo* protein folding, protein folding, cellular protein complex assembly, and protein polymerization (*p* = 9.6 × 10^–33^∼4.4 × 10^–22^). The top two functional subnetworks (Figure 4) were centered around six hubs, including three transcription factors (SMAD3, PAX6, UBF), two generic binding proteins (BLNK, MTS1) and one generic enzyme (HDC), in a high range of crosstalk (ranging from 5 to 14 interactions) with other genes (*p* = 3.25 × 10^–18^, Z-score = 21.93). These subnetworks contained 15 objects (spanned by 14 seed nodes) and 83 interactions (58 were activation and 25 were inhibition), which mainly involve in GO processes of positive regulation of biological process, cellular process, signal transduction, response to stimulus and macromolecule metabolic process (*p* = 3.7 × 10^–29^–8.8 × 10^–22^). In addition, 16 canonical pathways were presented on the subnetwork. The top three functional subnetworks (Figure 5) contained 16 objects (spanned by 14 seed nodes) and 64 interactions (49 were activation and 15 were inhibition), which have two hubs in a transcription factor (EGR1) and a GPCR receptor (FZD7) with a range from 5 to 18 interactions (*p* = 3.25 × 10^–18^, Z-score = 21.93). These subnetworks were involved with several GO processes including canonical and non-canonical Wnt receptor signaling pathways, a positive regulation of biological process and cellular process, and a signal transduction (*p* = 3.3 × 10^–35^–1.6 × 10^–29^). In addition, six canonical pathways were presented on the subnetwork.

**TABLE 3:** The top three biologically enriched subnetworks^a^.

No	Key subnetwork objects	GO processes	No. of seed nodes	No. of pathways	*p*-value^b^	Z-score^c^
1	DPP4, Ankyrin-B, ADNP, MCR, TAO2	“*de novo*” posttranslational protein folding (26.0%), “*de novo*” protein folding (26.0%), protein folding (31.5%), cellular protein complex assembly (28.8%), protein polymerization (19.2%)	15	0	2.33 × 10^−20^	24.35
2	BLNK, Granzyme B, PAX5, C3, Follistatin	positive regulation of biological process (80.0%), positive regulation of cellular process (76.0%), positive regulation of response to stimulus (46.7%), positive regulation of signal transduction (40.0%), positive regulation of macromolecule metabolic process (56.0%)	14	16	3.25 × 10^−18^	21.93
3	WNT4, TIMP2, GCKR(MAP4K5), B-Raf, SFRP1	canonical Wnt receptor signaling pathway (28.7%), positive regulation of biological process (82.5%), positive regulation of cellular process (77.5%), non-canonical Wnt receptor signaling pathway (20.0%), signal transduction (83.8%)	14	6	3.25 × 10^−18^	21.93

*^a^Results are based on 274 selected genes (seed nodes). ^b^p-value was calculated using hypergeometric test. ^c^Z-score was calculated based on MetaCore base knowledge.*

## Discussion

A rich and large-scale GWA data has been produced over past few years to document complex traits like BPD. The pathway-based analytics strategy provides an opportunity to uncover enriched pathways that are involved with the etiology of BPD based on prior knowledge of gene functions and molecular mechanisms. In this study, we reported 36 overrepresented pathways using a GWA dataset for BPD in GAIN, where genes in the same pathway were jointly associated with BPD. It is worth noting that many of these genes did not reach significant associations in GWA studies of BPD at a gene-level but reveal their potential roles in pathway-based analyses. In gene-level association analyses of the GAIN GWA study for BPD, the most genome-wide significant loci were found in *GRAMD1B* (rs4936819, *p* = 1.2 × 10^–6^), although it did not reach genome-wide significance threshold level at 5 × 10^–8^. Not surprisingly, we observed that many genes were included in multiple pathways to increase the risk of BPD but not reported in the GAIN GWA study for BPD. For example, *HTR3B* and *CACNA1C* (the top 6th and 12th in BPD genes) were included in substrate specific transmembrane transporter activity, substrate specific transporter activity, cation transmembrane transporter activity, ion transmembrane transporter activity, metal ion transmembrane transporter activity, gated channel activity, and cation channel activity (Kao et al., 2014). Evidence also supported that *HTR3B* encodes the subunit B of type 3 receptor for 5-hydroxytryptamine (serotonin). It was also found to be a susceptible gene for the development of BPD while *CACNA1C* was reported to be associated with the involvement of calcium channels in the biological mechanisms of BPD (Frank et al., 2004; Kloiber et al., 2012).

Among the 36 enriched pathways, we examined the degree of overlapping for significant genes (*p* < 0.05) in these pathways to evaluate their crosstalk. The resulting number and proportion of overlapping genes were shown in Supplementary Table 3. The proportion of significant genes (i.e., contain at least one SNP having *p* < 0.05) among all pathways was between 12 and 88% (average = 49.4%). This demonstrated that these significant pathways were dominated by many genes rather than one single gene. Our results exhibited a low to intermediate level of overlapping across pathways, indicating some crosstalk of molecules in enriched pathways. Among all pair-wise pathway comparisons, 48.4% did not have any significant genes overlapping, 33.6% had a low degree of overlapping (less than 20%), 8.6% had a moderate degree of overlapping (20–70%), and only 9.4% pathways had a high degree of overlapping (more than 70%). The fact that only a few genes were commonly identified in significant pathways for BPD further reflects the difficulty we faced in identifying “the genes” for complex diseases.

Many disease traits are usually caused by the dysfunction of several susceptible gene loci with small main and interaction effects. In fact, there may exist some (or even a few) key genes to dominate particular functions within a specific biological pathway. To capture this, we allowed such genes to play important roles relevant to BPD in the pathway. Thus, a weighting algorithm linking to prior biological knowledge was introduced into our analytic strategies for the pathway analysis. Genes with stronger prior information contributed majorly to the significance of the pathway. We found the number of enriched pathways increased with the proportion of significant genes (i.e., prior information) of a pathway. The development of pathway-based approaches that incorporate prior biological knowledge can identify novel disease susceptibility pathways along with “the key genes,” which will greatly facilitate the interpretation of GWA data biologically. Therefore, without highlighting the effects of these key genes in the pathway analysis, it is difficult to interpret their biological mechanisms correctly.

The *min-p* is a commonly used approach to assess association evidence at the gene-level in the pathway-based analysis. However, using the *min-p* statistic to represent the significance of a gene may be limited. For instance, if a number of markers within a gene region are moderately associated with a disease trait, the signal of such gene may be downweighed by not having “one” particular significant signal. Thus, combining all information of SNPs (i.e., *combined-p*) in a gene can aggregate the overall evidence that the gene-set association and SNPs with moderate effects can be included. Different strategies of defining the gene-level statistic may have substantial influences on results. This seems to be reasonable and also supported by observations evidence (Kao et al., 2012). One possible future direction in defining gene-level statistic is to adopt a mixed approach of using *min-p* and *combined-p*. With the mixed algorithm, an appropriate gene-level statistic will be computed to represent each gene properly.

In this current study, we found several BPD-susceptibility pathways were significantly related to metabolism that is not reported in previous studies using GWA SNPs data. However, there is overwhelming evidence to suggest that many metabolic pathways have been reported to be linked to complex traits, particularly psychiatric disorders (Saxena, 2009; Wood and Wood, 2013). In the past, a meta-analysis of metabolic abnormalities in BPD reported that bipolar patients, particularly patients of older age, are at a high risk for metabolic syndrome (Vancampfort et al., 2013). Priebe et al. (2012) used genome-wide SNP data to search for the presence of copy number variations in 291 early-onset bipolar patients and 872 healthy controls to implement pathways and biological processes. They found many pathways were significantly enriched in drug metabolism, lipid metabolism, and molecular transport, which were in line with our findings. Our results were also consistent with other studies based on using information from allele-specific gene methylation and incorporating information of microRNAs into the pathway analysis in the GAIN GWA study for BPD (Chuang et al., 2013; Shih et al., 2013). Besides, schizophrenia patients were found to be associated with thiol metabolism. In addition, abnormalities in metabolic cascades and metabolic disturbances were further observed in schizophrenia patients (Thakore, 2004; Kirkpatrick et al., 2008). Overall, the above evidence suggests that metabolic syndromes and complex psychiatric disorders like BPD appear to share some common in genetic factors, and may contribute to medical co-morbidity, including endocrine disturbances, dysregulation of sympathetic nervous system, and behavior patterns in these patients (Fagiolini et al., 2008). Our results identified nine enriched metabolic pathways that were significantly associated with BPD. These pathways were involved with human metabolic profiles, including drug, cofactors and vitamins, carbohydrate, lipid, and xenobiotics biodegradation. Importantly, human metabolizing systems act as a role of detoxification and transport through specialized enzymatic systems to aid excretion of xenobiotics, including drugs.

The BPD-related subnetworks (Figures 3–5 and Table 3) are complex and sophisticated, involving with several biological processes, cellular processes, signal transduction, metabolic processes, neuronal activities, immune system, and inflammation processes. The most significant subnetwork (Figure 3) is primarily related to the activation mechanism of transcription regulation between effects of *SP1* and many proteins (e.g., MAD, Prostacyclin receptor, *NOX5*, *LHX3*, PGE2R4, PKC-beta2, MCR, Claudin-1, p57 and IP3R1). This subnetwork plays a role in cell growth and apoptosis (e.g., *NOX5*), cell differentiation (e.g., *TCF7L1*, also known as *TCF3*), major transcript (e.g., Ankyrin-B), and ion or water transport (e.g., MCR). The second significant subnetwork (Figure 4) plays a role in regulating B-cell function and development (e.g., *BLNK*), B-cell differentiation and neural development (e.g., *PAX5*), immune system and inflammatory response (e.g., Granzyme B, *C3*), cellular proliferation and differentiation (e.g., Follistatin), and mediation of the control of cellular processes including cell cycle, neuron growth, ion channel regulation, and immune response (e.g., PKC). The third significant subnetwork (Figure 5) is central with two hubs (*EGR1* and *FZD7*). *EGR1* plays a critical role in animal models of maternal behavior on stress responses in the offspring (Weaver et al., 2004). The mechanism underlying the effect of early maternal behavior involves the EGR-mediated regulation of glucocorticoid receptor that may influence psychiatric illness susceptibility and abnormal anxiety-related behaviors later in life (Fish et al., 2004). McGowan et al. (2009) conducted a study in postmortem brains and suggested that similar mechanisms may occur in humans. *FZD7* was also identified to be associated with psychiatric or neurological disorders (Hoseth et al., 2018). This subnetwork plays a role in the response to environmental stress [e.g., *GCKR* (*MAP4K5*)], long-term memory (*ARC*), hippocampal neuron (B-Raf), and in regulating cell growth and differentiation (*SFRP1*). In this study, we identified 26 BPD-related functional subnetworks, which provide us an opportunity to facilitate future follow-up and functional studies for bipolar.

Many enriched pathways and selected genes were significantly associated with BPD in this study. Of which several genes and pathways were discussed and found to be consistent with previous studies. Particularly, six metabolic pathways (drug metabolism, retinol metabolism, pentose and glucuronate interconversions, porphyrin and chlorophyll metabolism, starch and sucrose metabolism, ascorbate and aldarate metabolism) were connected to dementia. A metabolic-caused dementia is a loss of function in the brain, e.g., cognitive changes and memory loss, that often occurs with certain psychiatric disorders like BPD. For instance, drugs are frequently a cause of dementia, which may impair cognition indirectly through metabolic effects (Starr and Whalley, 1994). Retinol metabolism was connected to an increased risk of dementia development. Retinol hypofunction and impaired transport may contribute to patients with memory impairment in Alzheimer’s disease (AD) and dementia (Goodman and Pardee, 2003). Two metabolic pathways, pentose and glucuronate interconversions (Zheng et al., 2019), and starch and sucrose metabolism (Ling et al., 2021) may play roles in learning and cognitive impairment that are caused by abnormal nitric oxide production and monoaminergic neurotransmitters in AD, BPD, and/or dementia patients. Other metabolisms, including porphyrin and chlorophyll metabolism (Wang et al., 2015), and ascorbate and aldarate metabolism (Chen et al., 2011) were biologically or molecularly connected with psychiatric disorders (e.g., AD, BPD) and dementia. We noticed that some of enriched network pathways that were not reported previously suggest that there may be potential links between BPD and the risk of dementia or possibly a chance association.

There are some limitations in our study. First, our pathway analysis relied on the accuracy and completeness of pathway annotation databases (e.g., MSigDB). Some genes may have potential impacts on BPD but not annotated in pathway databases, and they may be excluded from our analyses. Other datasets, such as IPA knowledge base,^4^ that provides detail-rich, highly structured knowledge for over 1,582,000 biological and chemical concepts in 19,635 humans, 15,194 mice, and 8,190 rat genes may be helpful to be considered in future analyses though their annotations need to be carefully selected. Second, it is possible that some genes might be falsely reported as significant loci in the literature. Thus, the accuracy of prior information is subjective to the completeness of data sources from the literature and current knowledge. We integrated gene information from different platforms or data sources to construct a combined score for each gene, followed by weighted pathway analysis to obtain more value-added pathway results using all existing genomic evidence and knowledge for BPD. Third, different strategies of defining the gene-level statistic may result in different outcomes in the pathway analysis. Some genes may be dominated by one (or a few) SNP(s) with a strong effect while other genes may be dominated by several SNPs with moderate effects. In this study, we only used the *min-p* statistic to extract information of SNPs for a gene. An advanced approach in calculating gene-level statistics for each gene is to extract SNPs information using both the *min-p* and the *combined-p* (e.g., random effects model or Bayesian statistical methods according to the structure of SNPs in a gene region (Stephens and Balding, 2009). Fourth, our study uses the signals of genetic association, while other genomic information (such as gene expression, gene regulation, etc.) has not been used yet. Concerning other useful genomic datasets, a possible utilization approach is to incorporate all possible genomic information into the pathway analysis. Finally, we only focused on Caucasian populations, using one GWA dataset for the pathway analysis and other for prior information collection (Kao et al., 2014). To generalize the results to the Eastern countries, a meta-analysis (or mega-analysis) of combining different populations (Caucasian and Han Chinese) of GWA data is underway to increase power to uncover the underlying biological mechanisms for BPD.

## Conclusion

Applying our comprehensive framework for the pathway and functional subnetwork analyses is useful for uncovering the underlying mechanisms and networks for complex traits. The evidence-based collection of prior information could benefit from quick accumulated data information and evidence from different aspects, which provides valuable information to quantify the contribution of genes in pathways for complex traits of interest. A number of novel genes that did not show significant associtions with BPD in the original single marker or gene analysis of GWA dataset were found to participate in several pathways, which, jointly with other genes, play roles in the pathogenesis of BPD. Although it remains largely unclear how the defect of pathways is specifically linked to the development of BPD, our identified pathways provided important biological insights into the interpretation of genome-wide association data for BPD. These findings are anticipated to facilitate future follow-up and functional studies for the connection and clinical implications between BPD and dementia.

## Data Availability Statement

The GWA data set was accessed through the Genetic Association Information Network (GAIN), database of Genotypes and Phenotypes (dbGaP) accession number phs000017.v3.p1 (http://www.ncbi.nlm.nih.gov/).

## Author Contributions

C-FK: study conception and design and acquisition of data. C-FK and WH: analysis and interpretation of data. C-FK, C-YK, T-YC, P-HK, WH, C-RC, Y-SL, and G-TY: draft and revise manuscript. All authors read and approved the final manuscript.

## Conflict of Interest

The authors declare that the research was conducted in the absence of any commercial or financial relationships that could be construed as a potential conflict of interest.

## Publisher’s Note

All claims expressed in this article are solely those of the authors and do not necessarily represent those of their affiliated organizations, or those of the publisher, the editors and the reviewers. Any product that may be evaluated in this article, or claim that may be made by its manufacturer, is not guaranteed or endorsed by the publisher.

## Footnotes

1
http://www.ncbi.nlm.nih.gov/

2
http://www.broadinstitute.org/gsea/msigdb/index.jsp

3
http://thomsonreuters.com/products_services/science/systems-biology/

4
http://www.ingenuity.com/products/ipa#/
