# Supplementary material for: Genetic Pathways and Functional Subnetworks for the Complex Nature of Bipolar Disorder in Genome-Wide Association Study
Source: Front Mol Neurosci. 2021 Nov 22;14:772584. doi: 10.3389/fnmol.2021.772584 (PMC8645771; doi:10.3389/fnmol.2021.772584)
Supplement: Supplementary file 2 [file Data_Sheet_1.pdf]

SUPPLEMENTARY MATERIAL

Supplementary Table 1. The number of significantly enriched pathways identified in permutation-based and probability-based approaches with/without weighting scheme

	Permutation-based			Probability-based	
	GSEA	Sum-statistic	Overlap[a]	Hypergeometric test	Overlap[b]
Non-weighting	68	12	12	10	3
Weighting	34	12	8	-	-
Overlap	22	12		-	

Abbreviation: Gene-Set Enrichment Analysis, GSEA.

[a]The number of overlap between GSEA and Sum-statistic among permutation-based methods.

[b]The number of overlap between permutation-based methods and probability-based method.

Supplementary Table 2. The remaining 23 significantly biological subnetworks[a] built by MetaCore

No	Key subnetwork objects	GO processes	No. of seed nodes	No. of pathways	*p*-value[b]	Z-score[c]
1	PLC-gamma 2, PAK3, RABGEF1, Rab-21, MUNC18-3	signaling (82.1%), single organism signaling (82.1%), cell communication (82.1%), signal transduction (75.6%), single-organism process (92.3%)	13	0	8.64e-17	20.74
2	DMRT1, UGT1A1, SOD3 (EC-SOD), C3a, UGT1A8	response to stimulus (98.7%), response to chemical stimulus (80.8%), cellular response to stimulus (89.7%), interferon-gamma-mediated signaling pathway (23.1%), T cell costimulation (21.8%)	12	1	2.66e-15	19.36
3	SLC5A1, KCNQ1, Fibulin-2, UGT1A4, UGT1A10	transferrin transport (32.9%), ferric iron transport (32.9%), phagosome maturation (32.9%), ATP hydrolysis coupled proton transport (30.3%), energy coupled proton transport, against electrochemical gradient (30.3%)	12	0	3.70e-15	19.09
4	MCT4, KIR2DS4, Rictor, HIF3A, IL-26	immune system process (68.8%), signal transduction (89.6%), cellular response to stimulus (94.8%), cell communication (92.2%), antigen processing and presentation of exogenous peptide antigen via MHC class I, TAP-independent (19.5%)	11	1	1.44e-13	17.45
5	HNF1-beta, CDK6, Kv2.1, PP2C alpha, iC3b	positive regulation of metabolic process (56.4%), positive regulation of cellular process (65.4%), positive regulation of biological process (67.9%), positive regulation of cellular metabolic process (52.6%), regulation of response to stimulus (56.4%)	11	1	2.23e-13	17.10
6	IKK-alpha, FNBP1, SLC6A15, WNT2, HIVEP2	positive regulation of macromolecule metabolic process (74.4%), positive regulation of transcription, DNA-dependent (61.5%), positive regulation of cellular metabolic process (74.4%), positive regulation of RNA metabolic process (61.5%), positive regulation of macromolecule biosynthetic process (62.8%)	11	0	2.23e-13	17.10
7	HIVEP2, Fibulin-2, TIMP2, NELL1, LAMA5	signal transduction (89.3%), intracellular signal transduction (58.7%), cell activation (46.7%), signaling (89.3%), single organism signaling (89.3%)	10	0	5.75e-12	15.70

8	Talin-1, PAK3, IFN-alpha, TCF7L1 (TCF3), SFRP1	positive regulation of biological process (83.3%), positive regulation of cellular process (78.2%), regulation of response to stimulus (69.2%), cellular response to stimulus (89.7%), regulation of signal transduction (60.3%)	10	1	6.56e-12	15.59
9	IKK-alpha, CCL1, Beta-arrestin1, PGE2R4, MAD1	regulation of multicellular organismal process (69.7%), positive regulation of cellular process (80.3%), response to organic substance (71.1%), cell surface receptor signaling pathway (76.3%), signal transduction (86.8%)	10	0	6.56e-12	15.59
10	GCKR(MAP4K5), CD53, DSC2, IFN-alpha, CDK5	regulation of signal transduction (74.4%), regulation of response to stimulus (80.8%), regulation of signaling (76.9%), positive regulation of cellular metabolic process (74.4%), positive regulation of biological process (88.5%)	10	0	7.46e-12	15.49
11	CD40(TNFRSF5), IFN-α,TCF7L1(TCF 3), SOCS6,CDK5	regulation of signal transduction (72.2%), regulation of response to stimulus (78.5%), positive regulation of macromolecule metabolic process (72.2%), positive regulation of metabolic process (73.4%), positive regulation of cellular process (83.5%)	10	0	7.46e-12	15.49
12	NALP7 (PYPAF3), BICC1, Adenylate cyclase type VI, IRS-1,IL-17 receptor	G-protein coupled receptor signaling pathway, coupled to cyclic nucleotide second messenger (33.8%), cell-cell signaling (54.5%), adenylate cyclase-inhibiting G-protein coupled receptor signaling pathway (22.1%), adenylate cyclase-modulating G-protein coupled receptor signaling pathway (27.3%), synaptic transmission (41.6%)	9	1	2.23e-10	13.88
13	Beta-arrestin1, DPY19L1, PAX5, 14-3-3 eta, FAZF	positive regulation of transcription, DNA-dependent (64.1%), positive regulation of gene expression (65.4%), positive regulation of RNA metabolic process (64.1%), regulation of cell differentiation (64.1%), positive regulation of macromolecule biosynthetic process (65.4%)	9	0	1.99e-10	13.97
14	IFN-alpha, FOXM1, PGE2R4, MLL4, HNF1-beta	positive regulation of biological process (92.4%), positive regulation of cellular process (89.9%), positive regulation of cellular metabolic process (77.2%), response to organic substance (79.7%), positive regulation of metabolic process (77.2%)	9	0	2.23e-10	13.88
15	14-3-3 eta, IFNA7, NALCN, IFNGR1, IFN-alpha	response to organic substance (67.9%), single-organism process (96.4%), response to stress (67.9%), type I interferon-mediated signaling pathway (20.2%), response to stimulus (92.9%)	9	8	3.49e-10	13.51

16	IRS-1, IKK-alpha, TCF7L1 (TCF3), STK4, PAX5	enzyme linked receptor protein signaling pathway (65.8%), regulation of response to stimulus (88.6%), positive regulation of response to stimulus (69.6%), cell surface receptor signaling pathway (89.9%), positive regulation of biological process (97.5%)	8	0	5.91e-09	12.26
17	NCOA1 (SRC1), SWAP-70, NAT-1, DPP4, IFNA1	regulation of response to stimulus (76.6%), cellular response to chemical stimulus (68.8%), response to stress (79.2%), positive regulation of biological process (84.4%), response to organic substance (72.7%)	8	0	5.91e-09	12.26
18	CDK5, FOXM1, HSP90 alpha, CDK6, TCF7L1 (TCF3)	positive regulation of cellular process (82.9%), positive regulation of macromolecule metabolic process (71.1%), positive regulation of metabolic process (75.0%), positive regulation of cellular metabolic process (71.1%), cellular response to stimulus(94.7%)	7	0	1.37e-07	10.65
19	IRS-1, IFNA1, IL-2, BAG-1, CTIP2	7-methylguanosine mRNA capping (20.9%), 7-methylguanosine RNA capping (20.9%), RNA capping (20.9%), transcription-coupled nucleotide-excision repair (20.9%), regulation of nucleobase-containing compound metabolic process (73.1%)	6	0	1.24e-06	9.75
20	ATF-3, DPP4, IFN-alpha, IFNA7, PTPR-beta	cellular response to chemical stimulus (72.2%), response to organic substance (74.7%), positive regulation of cellular process (83.5%), cellular response to organic substance (63.3%), positive regulation of biological process (84.8%)	6	0	2.54e-06	9.11
21	PKC-epsilon, NOL3, DAP12, TRIM, IRS-1	regulation of immune system process (61.3%), positive regulation of response to stimulus (64.0%), immune system process (68.0%), positive regulation of biological process (85.3%), regulation of response to stimulus (74.7%)	6	0	2.73e-06	9.04
22	SLC2A13, TTC39B, FGF5, TRIO, RelA (p65 NF-kB subunit)	viral genome expression (46.6%), viral transcription (46.6%), translational termination (46.6%), cellular protein complex disassembly (46.6%), SRP-dependent cotranslational protein targeting to membrane (46.6%)	5	0	3.83e-05	7.60
23	CD40(TNFRSF5), IKK-α, HSP90 α, PKC-delta, CBP	cell surface receptor signaling pathway (88.3%), signal transduction (97.4%), enzyme linked receptor protein signaling pathway (59.7%), cell communication (98.7%), signaling (97.4%)	4	0	6.39e-04	5.82

Note: [a]Restults are based on 274 selected genes (seed nodes). [b]*p*-value was calculated using hypergeometric test. [c]Z-score was calculated based on MetaCore base knowledge.

Supplementary Table 3. The number and proportion of overlapping genes across pathways

Pathway	n_{GWA}^{pw} / n^{pw}	pp^{sig}	No. of overlap genes among pair-wise pathway comparisons in different degree of overlap[a]			
			Non 0	Low (0,0.2]	moderate (0.2,0.7]	high (0.7,1]
**KEGG:**						
Drug metabolism other enzymes	48/51	0.52	18	5	11	1
Retinol metabolism	60/64	0.55	20	4	10	1
Pentose and glucuronate interconversions	25/28	0.60	19	5	8	3
Porphyrin and chlorophyll metabolism	37/41	0.49	16	8	10	1
Starch and sucrose metabolism	49/52	0.53	17	7	8	3
Ascorbate and aldarate metabolism	23/25	0.61	18	6	8	3
Drug metabolism cytochrome P450	68/72	0.46	18	6	8	3
Metabolism of xenobiotics by cytochrome P450	68/70	0.47	16	8	8	3
Steroid hormone biosynthesis	51/55	0.55	17	7	10	1
**GO:**						
Extracellular region part	313/332	0.46	21	13	0	1
Extracellular space	224/239	0.45	21	13	0	1
Ion transport	174/184	0.49	15	11	5	4
Substrate specific transmembrane transporter activity	321/341	0.47	13	13	0	9
Substrate specific transporter activity	366/388	0.46	11	15	0	9
Cytosol	191/202	0.40	15	20	0	0
Cation transmembrane transporter activity	201/211	0.55	16	10	1	8
Ion transmembrane transporter activity	259/275	0.53	15	11	0	9
Metal ion transmembrane transporter activity	140/145	0.56	19	7	1	8
Voltage-gated channel activity	70/73	0.63	21	5	1	8
Gated channel activity	115/121	0.57	20	6	1	8
Voltage-gated cation channel activity	64/66	0.62	21	5	0	9
Cation channel activity	115/118	0.56	21	5	1	8
**REACTOME:**						
Glucuronidation	17/19	0.59	21	3	0	11
Phase II conjugation	56/60	0.46	19	5	9	2
Purine ribonucleoside monophosphate biosynthesis	11/11	0.18	33	1	1	0
Biological oxidations	120/127	0.45	16	8	7	4
**Curated gene-set:**						
Riggi ewing sarcoma progenitor UP	375/426	0.50	7	28	0	0
Zhang breast cancer progenitors UP	360/448	0.36	9	26	0	0

Mullighan mll signature 1 UP	351/389	0.38	14	21	0	0
Weber methylated icp in fibroblast	16/16	0.12	34	1	0	0
Rizki tumor invasiveness 3D DN	215/234	0.38	9	26	0	0
Bonci targets of MIR15A and MIR16_1	81/81	0.43	13	22	0	0
Mccabe bound by HOXC6	350/461	0.40	3	32	0	0
Martinez response to trabectedin	40/42	0.88	23	12	0	0
Manalo hypoxia UP	191/210	0.57	17	18	0	0
Onder CDH1 targets 2 UP	226/258	0.56	4	31	0	0
Number of crosstalk in different categories			***305***	***212***	***54***	***59***
Percentage of crosstalk in different categroies			***48.4***	***33.6***	***8.6***	***9.4***

Abbreviation: n^{pw}, the number of genes in pathway; n_{GWA}^{pw}, the number of genes on chip; pp^{sig}, proportion of significant genes.

[a] The number and the percentage of overlap genes among pair-wise pathway comparisons in different degree of overlap e.g. non-overlap, low degree (0, 0.2], moderate degree (0.2, 0.7], and high degree (0.7, 1].

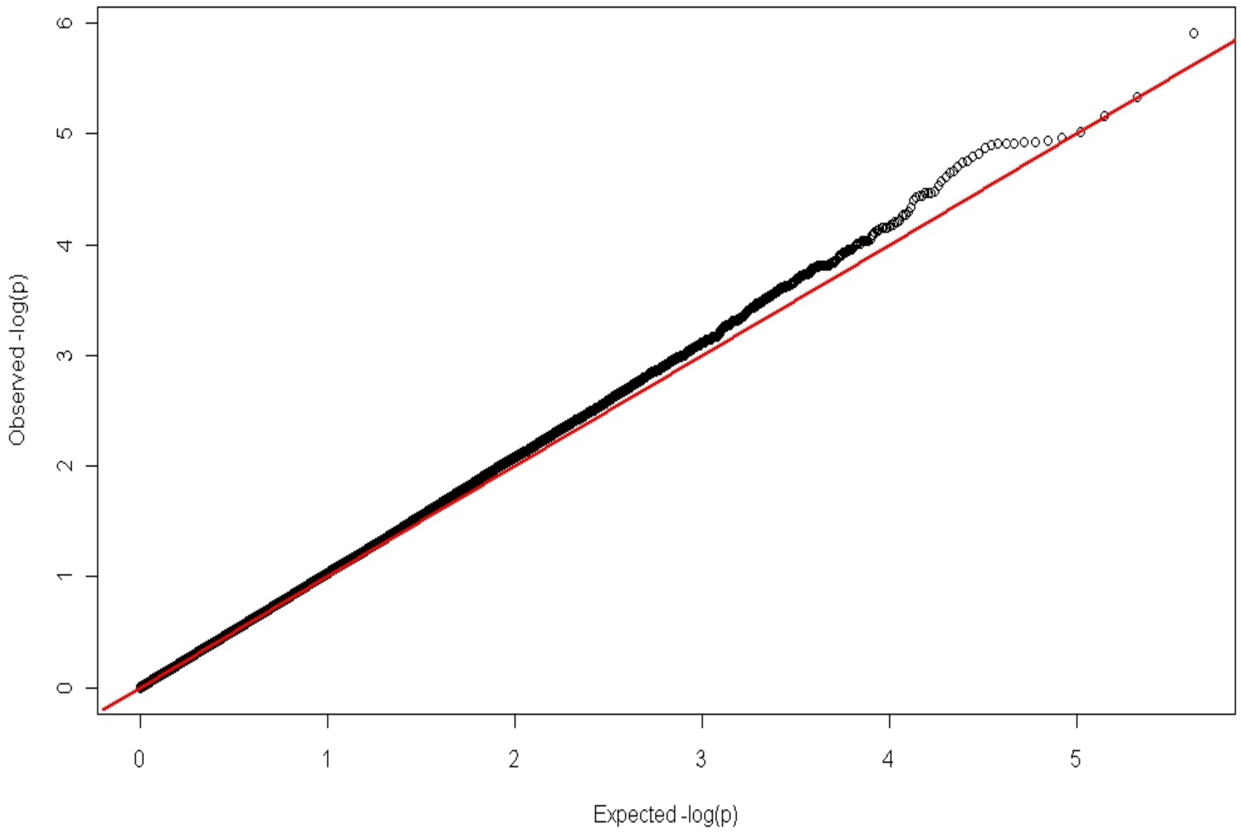

Supplementary Figure 1. The quantile-quantile plot of all analyzed SNPs in a genome-wide association (GWA) data for BPD in GAIN.

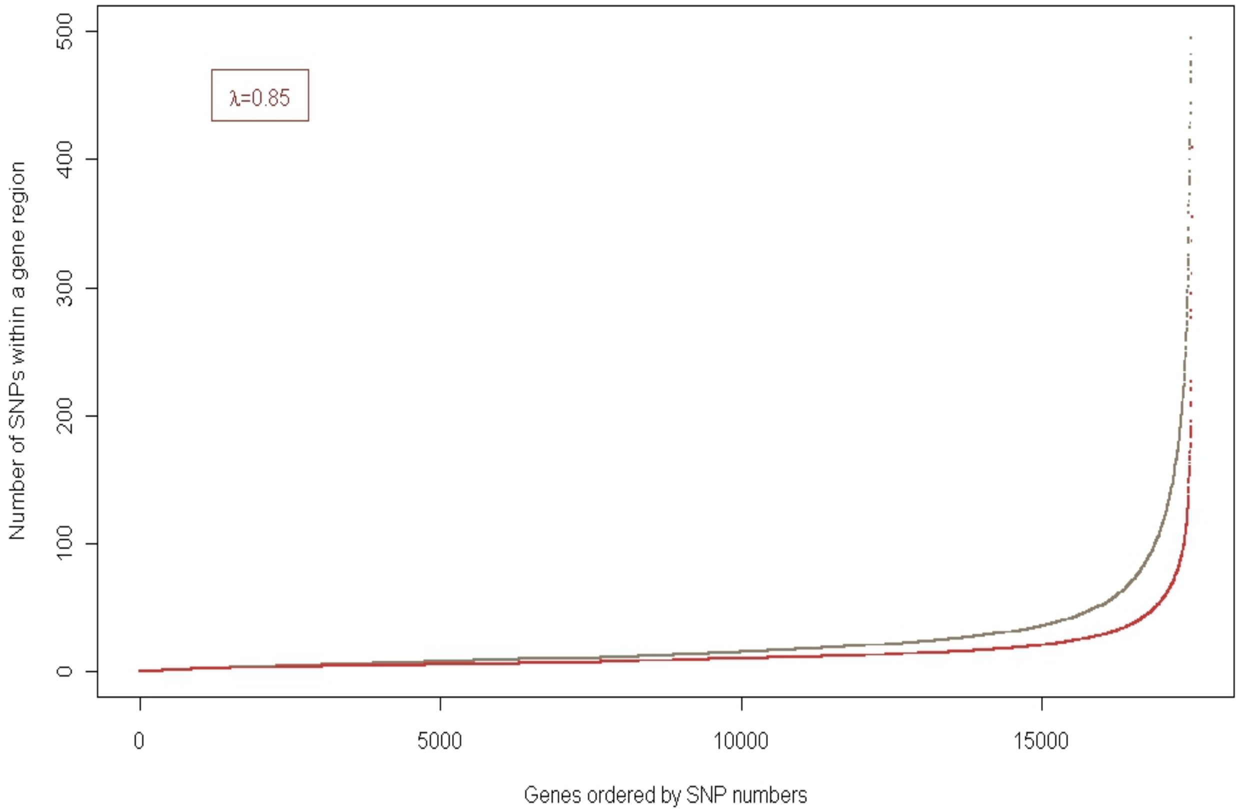

Supplementary Figure 2. Corrected SNP numbers within a gene region in a genome-wide association (GWA) data for BPD in GAIN.
